# Psychological distress during the COVID-19 pandemic in Ethiopia: an online cross-sectional study to identify the need for equal attention of intervention

**DOI:** 10.1186/s12991-021-00344-4

**Published:** 2021-03-25

**Authors:** Argaw Ambelu, Zewdie Birhanu, Yimenu Yitayih, Yohannes Kebede, Mohammed Mecha, Jemal Abafita, Ashenafi Belay, Diriba Fufa

**Affiliations:** 1 Department of Environmental Health Sciences and Technology, Faculty of Public Health, Jimma University, Jimma, Ethiopia; 2 Departemnt of Health, Behavior, and Society, Faculty of Public Health, Jimma University, Jimma, Ethiopia; 3 Department of Psychiatry, Faculty of Medical Sciences, Jimma University, Jimma, Ethiopia; 4 Department of Internal Medicine, Faculty of Medical Sciences, Jimma University, Jimma, Ethiopia; 5 Department of Economics, College of Business and Economics, Jimma University, Jimma, Ethiopia; 6 Department of English Language and Literature, College of Social Sciences, Jimma University, Jimma, Ethiopia; 7 Department of Paediatrics and Child Health, Faculty of Medical Sciences, Jimma University, Jimma, Ethiopia

**Keywords:** COVID-19, Ethiopia, Online survey, Pandemic, Psychological distress

## Abstract

**Background:**

The COVID-19 pandemic led individuals to suffer from different levels of mental health problems such as psychological distress, anxiety, depression, denial, panic, and fear. This study aimed at determining the prevalence of psychological distress and associated factors among the Ethiopian population during the COVID-19 pandemic.

**Methods:**

A cross-sectional study was performed through an online survey using different online platforms. The questionnaire was created through Google Form and the survey link was administered by e-mail, LinkedIn, Telegram, and Facebook. Educated Ethiopian population who have access to the internet were invited to participate through an online survey and addressed to 929 respondents. The study participants completed the survey anonymously without any personal identifier. The psychological distress was assessed using the Kessler 10-item tool to measure psychological distress. Data were analyzed using SPSS and logistic regression to examine mutually adjusted associations, expressed as adjusted odds ratios. A generalized additive model was also employed to identify additional predictors using R.

**Results:**

The prevalence of high psychological distress among the study population was 236 (25.5%). Of all respondents, 421 (45.1%) had low psychological distress, 274 (29.4%) had moderate psychological distress, 164 (17.6%) had high psychological distress, and 72 (7.3%) had very high psychological distress. Psychological distress increased with being at young and middle-aged adults, getting information from social media, and not correctly practicing infection prevention and control measures to prevent COVID-19 infection. Respondents with high perceived severity had increased psychological distress. On the contrary, those with the highest score of perceived response efficacy had low distress.

**Conclusion:**

Prevalence of psychological distress was substantial. The need for intervention of psychological distress inline with the prevention of COVID-19 is critically essential. The intervention target groups are those whose information sources are from social media, young and middle-aged adults, and those who do not correctly practice infection prevention and control measures against COVID-19 infection.

## Introduction

The 2019 coronavirus disease (COVID-19) pandemic is registered as the largest outbreak of atypical pneumonia since the severe acute respiratory syndrome (SARS) outbreak in 2003 [1]. On Jan 30, 2020, WHO declared the current novel COVID-19 as pandemic disease and a Public Health Emergency of International Concern posing a high risk to countries with vulnerable health systems [2]. The disease has impacted the economic, social and health of all nations where the magnitude differs by geography, society and country. It is registered as a global public health emergency due to its rapid transmission, an increment of the confirmed case, and high mortality [3]. It is highly contagious and transmits to humans through respiratory droplets, body and surface contacts [4]. The contagious nature of the disease, the stigma it brings, economic pressures, isolation of the infected individuals, and panic/fear of the disease leads the public to other health problems.

Psychological distress is one of the major public health problems that may occur as a result of the work environment [5] and different local and global incidents, like the COVID-19 pandemic. COVID-19 became a major concern for global health [6]. The outbreak of COVID-19 in Ethiopia was officially recognized on 13 March 2020, after a Japanese national arrived in Ethiopia from his Burkina Faso trip, tested positive for the novel COVID-19. From this time onwards, there was a surge of cases, with a peak of 494 new infections recorded as of May 24 and five deaths had occurred, and as well as there are several exposed individuals who are under quarantine. The emergency committee has stated that the spread of the COVID-19 pandemic may be interrupted by staying at home, quarantine, alongside city lockdown, school closure, early detection, prompt treatment, and the implementation of a robust system to trace contacts [7]. Such, health emergency measures to control the spread of the COVID-19 disease had a strong influence on the psychological health of the population. Separation from loved ones, the loss of freedom due to different restrictions, uncertainty over disease status, and boredom can, on occasion, create dramatic adverse effects on mental health [8]. Feeling isolation can lead to poor sleep, psychological distress, anxiety, depressive symptoms, and impaired executive function. When executing tasks of the brain are impaired, individuals had more difficultly to focus on issues, manage their emotions, fail to remember information, which leads to mental illness [9].

Furthermore, suicide has been reported [10], substantial anger generated, and complaints brought following the imposition of emergency health measures in outbreaks [11]. In the reviewed studies, the financial loss as a result of emergency health measures created serious socioeconomic distress [12] and was found to be a risk factor for symptoms of psychological disorders [13]. The study revealed that the risk of contracting or carrying the virus could provoke substantial acute stress disorder, depression, post-traumatic stress disorder, insomnia, irritability, and emotional exhaustion [8].

Despite this, there is no information available regarding the psychological impact of the COVID-19 pandemic in Ethiopia. While many resources are devoted to biomedical research and medical treatment, psychological problems of the COVID-19 pandemic are mainly ignored in the world, particularly in Ethiopia. Although emergency health measures during the COVID-19 pandemic has been adopted for protecting physical health from infectious diseases, it is crucial to consider the mental health implications of such emergency health measures.

Therefore, this study aimed at determining the prevalence of psychological distress and to identify associated factors among the Ethiopian population in response to the COVID-19 pandemic. The finding of this study will indicate the need and attention for psychological interventions while treating COVID-19 patients and disseminating disease prevention mechanisms. The study emphasizes the need for equal attention of intervention to psychosocial distress in the combat against the pandemic.

## Methods

### Study design and period

This cross-sectional study was performed through an online survey using different online platforms. The questionnaire was created through Google Form and the survey link was administered by e-mail, Telegram Facebook, LinkedIn, and Facebook page of Jimma University to assess psychological distress during COVID-19 pandemic. The questionnaire was available online for 2 weeks, from April 22 to May 4 2020. During that time, we tracked the completion of questionnaires, observing the date and time of the survey end.

### Study population

The literate Ethiopian population who have access to the internet were invited to participate in the study by responding to the online survey. The online survey was open until at least 900 respondents are responding to the online questionnaire assuming the sample size for the infinite population was satisfied. Respondents were included in the survey if they live in Ethiopia and have, at least, college-level training. Respondents permanently living outside Ethiopia were not invited to complete the survey. In 2 weeks, 929 respondents completed the questionnaire, and the survey was closed.

### Measurements

The questionnaire consisted: socio-demographic characteristics, the practice of infection prevention techniques of COVID-19, and psychological distress. Demographic variables included age, gender, marital status, education, occupation, and current place of residency.

The Kessler 10 (K10) tool was used to measure the psychological distress experienced by subjects during the last 4 weeks preceding the survey [14]. Respondents were instructed that the items constituted a list of ways they may have felt or behaved in the previous 4 weeks, and they scored on a scale of 1–5 depending on how frequently each symptom is experienced, where 1 = ‘none of the time’, and 5 = ‘all of the time’.

The K10 has ten items with a Likert rating scale ranging from 1 (not at all) to 4 (extremely). The full assessment scale contains ten items (scored from 0 to 50) with confirmed reliability and validity that measures psychological distress across diverse cultural settings. Thus, a minimum score of 10 indicates no psychological distress, and a maximum score of 50 indicates a severe level of psychological distress. The final K10 score was categorized into four levels: low psychological distress (10–15 score), moderate psychological distress (16–21 score), high psychological distress (22–29 score), and very high psychological distress (30–50 score) [15]. The Cronbach’s alpha was 0.89 for this study indicates the acceptable internal consistency of the scale used to measure psychosocial distress.

The total K10 scores of 22 or greater signify high psychological distress (high + very high level of psychological distress), whereas scores of 21 or less indicate low psychological distress (low + moderate level of psychological distress). Scores from the K10 are indicative of the levels of intervention, with 'very high' psychological distress scores (> 30) associating with a case for a mental disorder, and high scores are strongly associated with a current diagnosis of anxiety and depression using the Composite International Diagnostics Interview (CIDI) [16].

### Statistical analysis

The data were extracted, edited, and analyzed using SPSS version 23 for Windows. Frequency tables were used to summarize socio-demographic characteristics and prevalence of psychological distress. Bivariate logistic regression was performed separately for each independent variable. Independent variables with a *p*-value < 0.25 were entered into the final model for multivariable analysis. Variables in the mutually adjusted multivariable model with a two-sided *p*-value < 0.05 were considered statistically significant. Also, dimension reduction was made to bring multiple similar variables into one-component score using factor analysis. From our previous analysis, variables involved at different levels of perception, such as perceived response efficacy (PRE), perceived self-efficacy—personal level (PSE), perceived vulnerability (PV), perceived collective efficacy (PCE), and perceived severity/seriousness (PS), were identified. The first principal component scores were used to predict psychosocial distress. Similarly, component scores of participant’s trust in information sources, level of their knowledge, and their practice to prevent coronavirus were also used to predict psychological distress. A variable with the highest communalities (> 0.8) was removed to get component scores with the highest percent variance. The first component score, with a variance of 25% or higher, was accepted to represent the group variable. A generalized additive model (GAM) was fitted to identify the predictors of psychological distress among the scholarly communities of Ethiopia.

### Ethical clearance and consent to participate

The online survey was conducted after ethical clearance was obtained from the Ethical Review Board of the Jimma Institute of Health. Participants were informed to fill the online questionnaire voluntarily with a full right not to answer all or any of the questions. The survey was fully on a voluntary basis and no incentive or compensation was offered for their participation. The right of the participants to not fill all or part of the survey was clearly mentioned. The online survey has no personal identifier, so anonymity was maintained.

## Results

In total, 929 respondents completed the online survey. Of these, 834 (89.2%) were male, and 101 (10.8%) were female. A total of 314 (33.6%) of the respondents were aged from 30 to 34 years. More than half of the respondents, 494 (52.8%), had MSc or MA in educational qualification. The majority of the respondents were ever married 609 (65.1%) and of residency in the Oromia region 531 (56.8%). Just under half of the respondents, 419 (44.8%) were orthodox Christina by religious followers. More than half of the participants, 505 (54%) were university employees, while only 15 (1.6%) of respondents had no job (see Table 1).

**Table 1:** Distribution of psychological distress in relation to the socio-demographic characteristics of respondents among the Ethiopian population, 2020

Variable	*N* (%)	Psychological distress	
		No	Yes
Age groups (years)			
18–24	60 (6.4%)	51 (7.3%)	9 (3.8%)
25–29	225(24.2%)	158 (23.0%)	66 (27.7%)
30–34	313 (33.7%)	232 (33.3%)	82 (34.5%)
35–39	159 (17.1%)	117 (17.1%)	41 (17.2%)
40–44	81 (8.7%)	60 (8.6%)	23 (9.7%)
45–49	49 (5.3%)	35 (5.3%)	12 (5.0%)
50 and above	42 (4.5%)	38 (5.5%	5 (2.1%)
Sex			
Female	101 (10.9%)	70 (10.6%)	27 (11.3%)
Male	828 (89.1%)	617 (89.2%)	210 (88.7%)
Marital status			
Single	326 (35.1%)	246 (35.5%)	80 (33.6%)
Ever married	603 (64.9%)	445 (64.5%)	158 (66.4%)
Educational qualification			
Diploma^a^	26 (2.8%)	21 (3.1%)	5 (2.1%)
BSc/BA	167 (18.0%)	112 (16.1%)	55 (23.1%)
MSc/MA	493 (53.1%)	371 (53.6%)	122 (51.3%)
MD-GP	66 (7.1%)	50 (7.2%)	16 (6.7%)
MD-resident	43 (4.6%)	34 (4.9%)	9 (3.8%)
Ph.D	94 (10.1%)	70 (10.3%)	24 (10.1%)
MD-specialist	40 (4.3%)	33 (4.8%)	7 (2.9%)
Occupation			
Student	72 (7.8%)	58 (8.5%)	14 (5.9%)
No job	15 (1.6%)	12 (1.7%)	3 (1.3%)
Self and private employed	132 (14.2%)	91 (13.3%)	41 (17.2%)
Health workers	209 (22.5%)	150 (21.7%)	59 (24.8%)
University employed	501 (53.9%)	380 (54.8%)	121 (50.8%)
Region of residence			
Oromia	526 (56.6%)	398 (57.1%)	133 (55.9%)
Addis Ababa and Dire Dawa	150 (16.1%)	116 (16.6%)	34 (14.3%)
SNNPR	103 (11.1%)	69 (9.9%)	34 (14.3%)
Amhara	52 (5.6%)	36 (5.2%)	16 (6.7%)
Tigray	49 (5.3%)	38 (5.5%)	11 (4.6%)
Harari	24 (2.6%)	23 (3.3%)	1 (0.4%)
Other	25 (2.7%)	17 (2.4%)	9 (3.8%)
Religion			
Orthodox christian	417 (44.9%)	320 (46.2%)	97 (40.8%)
Muslim	114 (12.3%)	89 (12.9%)	25 (10.5%)
Protestant	336 (36.3%)	234 (34.0%)	102 (42.9%)
Wakeffeta	40 (4.3%)	31 (4.4%)	9 (3.8%)
Others	22 (2.4%)	17 (2.4%)	5 (2.1%)

^a^Respondents with grade 10 + but not obtained university degree

### Means and source of information

**Fig. 1:**
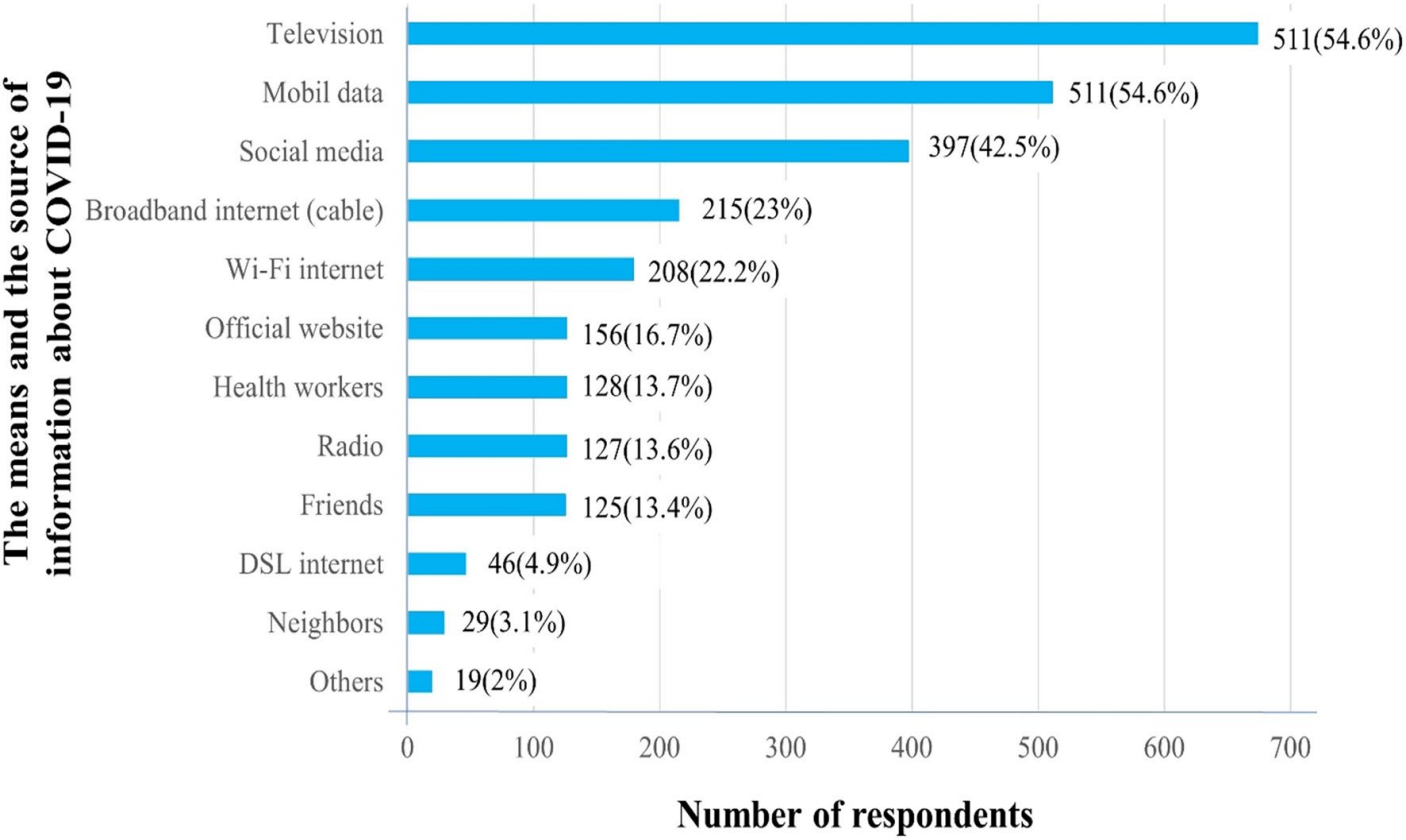
Distribution of means of information sources among Ethiopians, 2020

Respondents were asked to tick the top two information sources about the pandemic. The majority of respondents (72.5%) got different information about COVID-19 from television, followed by mobile (cellular) data internet (54.4), which is the only mobile service provider in the country. Figure 1 demonstrates the number of respondents using different sources of coronavirus information.

### The prevalence of psychological distress

The prevalence of high psychological distress among the Ethiopian population-based on 10-item Kessler psychological distress scale score ≥ 22 was 238 (25.5%). Of all respondents, 422 (45.1%) had low psychological distress, 275 (29.4%) had moderate psychological distress, 165 (17.6%) had high psychological distress, and 73 (7.3%) had very high psychological distress. High prevalence of psychological distress recorded was among males (88.7%, 95% CI 84.0–92.4) compared to females (11.3%, 95% CI 7.6–16.0). Of participants 30–34 years (66.4%, 95% CI 26.9–29.8) and those ever married in marital status (66.4%, 95% CI 60.5–72.7) reported the highest prevalence of psychological distress. On the other hand, the psychological distress prevalence was higher among University employees (50.8%, 95% CI 44.5–57.1) and those who were health workers (24.8%, 95% CI 19.3–30.3), compared to self or private employed, no job and student.

A total of 1.4% and 3.1% of participants had a hopeless feeling all the time and most of the time, respectively. In our study, the participants had a sense of nervousness all the time (2.8%), most of the time (5.9%), and sometimes (19.8%). Of participants, 1.2% had depressed feeling all the time, 4.4% had a depressed feeling most of the time and 16.4% had reduced feelings sometimes (Table 2).

**Table 2:** Responses of participants for the Kessler 10 questionnaire among the literate Ethiopian population during COVID-19 pandemic, 2020

Items	All of the time, N (%)	Most of the time, N (%)	Some of the time, N (%)	A little of the time, N (%)	None of the time, N (%)
Tired out for no good reason	28 (3.0%)	72 (7.7%)	203 (21.7%)	231 (24.7%)	401 (42.95%)
Feeling nervous	26 (2.8%)	55 (5.9%)	185 (19.8%)	286 (30.6%)	383 (41.0%)
Feeling so nervous that there is nothing calm you down	21 (2.2%)	21 (2.2%)	76 (8.1%)	145 (15.5%)	667 (71.3%)
Feeling hopeless	13 (1.4%)	29 (3.1%)	103 (11.0%)	173 (18.5%)	617 (66.0%)
Feeling restless or fidgety	9 (1.0%)	51 (5.5%)	103 (11.0%)	263 (28.1%)	509 (54.4%)
Feeling so restless that you could not sit still	10 (1.1%)	28 (3.0%)	81 (8.7%)	160 (17.1%)	656 (70.2%)
Feeling depressed	11 (1.2%)	41 (4.4%)	153 (16.4%)	307 (32.8%)	423 (45.2%)
Feeling that everything was an effort	122 (13.0%)	181 (19.4%)	210 (22.5%)	194 (20.7%)	228 (24.4%)
Feeling so sad that nothing could make cheer you up	10 (1.1%)	43 (4.6%)	144 (15.4%)	236 (25.2%)	502 (53.7%)
Feeling worthless	21 (2.2%)	25 (2.7%)	117 (12.5%)	161 (17.2%)	61,165.3

**Fig. 2:**
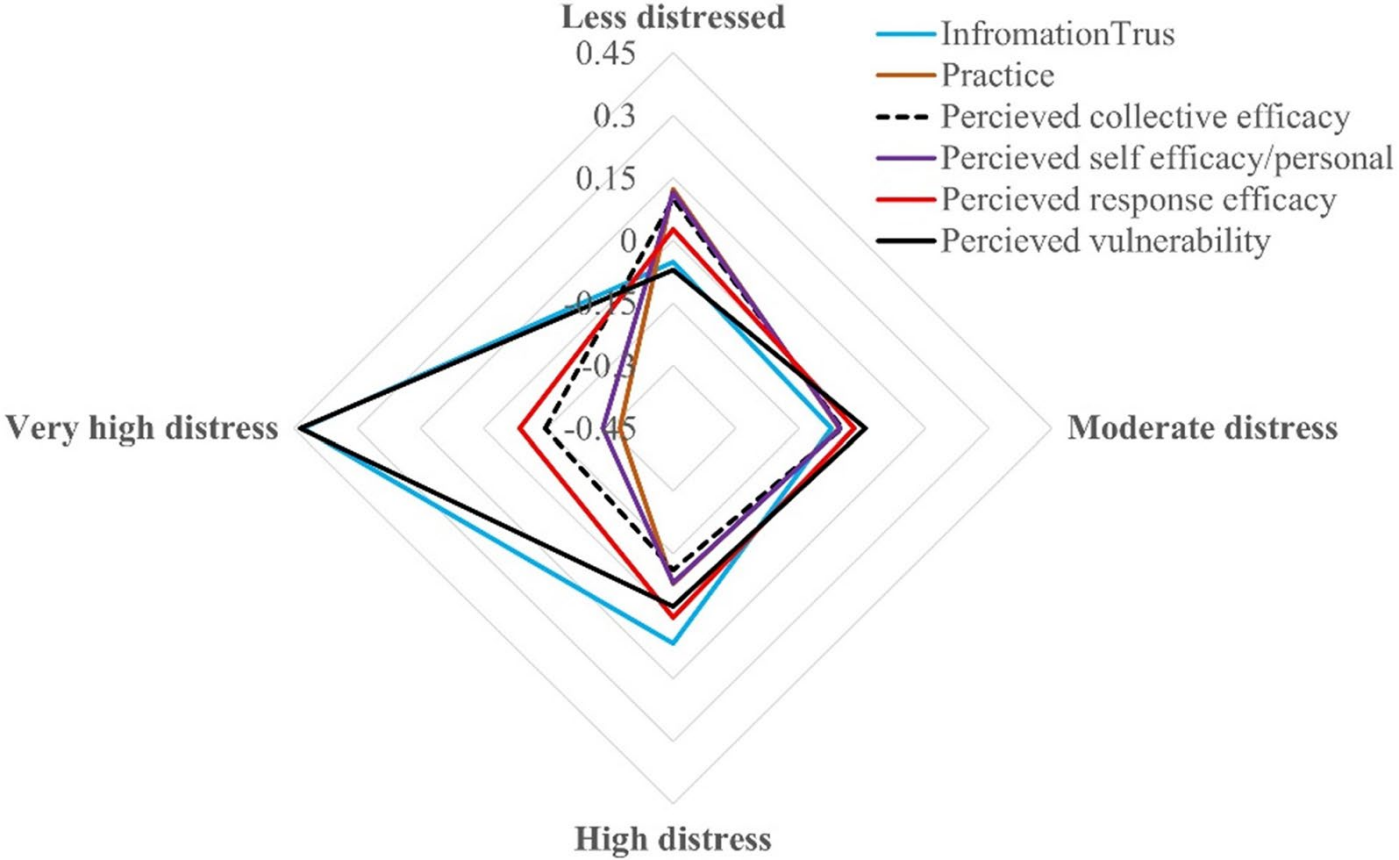
The distribution of different variables into four dimensions of the psychological distress among literate Ethiopians, May 2020

The distribution of different variables against the four categories of the psychological status of the respondents indicated that those who trust information sources are under very high psychological distress. Those respondents who were knowledgeable about coronavirus transmission and prevention have either moderate or no psychological distress (Fig. 2).

### Multivariable analysis

The multivariate logistic regression revealed that the odds of psychological distress was higher among participants age of 25–29 (AOR: 3.21; 95% CI 1.03–10.00), 30–34 years ((AOR: 3.31; 95% CI 1.10–10.01), 35–39 years (AOR: 3.42; 95% CI 1.12–10.41), and 40–44 years (AOR: 4.27; 95% CI 1.35–13.56) and in comparison with those the age of 50 years and above.

The likelihood of having psychological distress was highest among those who disagree on washing their hands frequently with water and soap to prevent COVID-19 infection (AOR 4.17, 95% CI 1.43–12.15) compared to their counterparts. Compared to the rest of the study participants, those who get information from social media had significantly higher odds of having psychological distress (AOR 1.42, 95% CI 1.02–1.99).

Those who disagree on having the resource (water, soap) to wash their hands (AOR: 2.62; 95% CI 1.20–5.70) were also found to have higher odds of psychological distress. It was also revealed that participants who disagree on having the skill to follow recommended handwashing practices to prevent COVID-19 infection were found to have higher odds of psychological distress when compared to their counterparts (AOR 5.39, 95% CI 1.17–24.87) (see Table 3).

**Table 3:** Independently associated factors with psychological distress among the Ethiopian population during COVID-19 pandemic, 2020

Variable	COR	95% CI	*p*-value	AOR	95% CI	*p*-value
Age groups						
18–24 25–29 30–34 35–39 40–44 45–49 50 and above	1.34 3.13 2.69 2.62 2.91 2.46 1	0.42–4.33 1.18–8.32 1.02–7.06 0.96–7.10 1.02–8.32 0.79–7.69	0.623 0.022 0.045 0.059 0.046 0.120	1.17 3.21 3.31 3.42 4.27 3.17 1	0.28–4.82 1.03–10.00 1.10–10.01 1.12–10.41 1.35–13.56 0.90–11.18	0.826 0.045* 0.034* 0.030* 0.014* 0.072
Sex						
Male Female	0.93 1	0.58–1.48	0.755	0. 77 1	0.45–1.31	0. 342
Educational qualification						
Diploma BSc/BA MSc/MA MD-GP MD-resident Ph.D MD-specialist	1.10 2.38 1.59 1.55 1.29 1.60 1	0.31–3.92 0.99–5.72 0.69–3.68 0.58–4.18 0.43–3.85 0.63–4.07	0.878 0.052 0.277 0.382 0.653 0.327	1.89 2.11 1.36 1.47 1.20 1.79 1	0.43–8.32 0.76–5.85 0.52–3.52 0.46–4.65 0.34–4.28 0.60–5.33	0.398 0.152 0.528 0.514 0.773 0.295
Occupation						
Student No job Self and private employed Health workers University employed	0.77 0.79 1.43 1.23 1	0.41–1.42 0.22–2.86 0.94–2.18 0.86–1.77	0.398 0.723 0.096 0.261	0.96 0.78 1.22 0.99 1	0.42–2.20 0.18–3.43 0.73–2.06 0.61–1.60	0.925 0.743 0.447 0.957
Current place of residence						
District town Zonal town Regional town Administrative city	1 0.70 0.67 0.74	0.43–1.14 0.38–1.18 0.42–1.30	0.157 0.163 0.290	1 0.82 0.79 0.93	0.46–1.45 0.40–1.56 0.48–1.78	0.495 0.499 0.822
Getting information from social media						
No Yes	1 1.31	0.98–1.77	0.070	1 1.42	1.02–1.99	0.039*
Getting information from health care workers						
No Yes	0.66 1	0.45–0.99	0.044	0.71	0.45–1.12	0.146
I am confident that I can wash my hands frequently with soap and water						
Strongly disagree Disagree Neither agree or disagree Agree Strongly agree	0.61 4.57 1.70 1.79 1	0.07–5.00 2.07–10.11 0.87–3.31 1.30–2.46	0.642 0.001 0.120 0.001	0.19 4.17 1.56 1.43	0.01–5.08 1.43–12.15 0.65–3.79 0.91–2.24	0.321 0.009* 0.319 0.117
Have the resource (water, soap) to wash my hands						
Strongly disagree Disagree Neither agree or disagree Agree Strongly agree	2.30 3.51 1.36 1.17 1	1.07–4.94 1.99–6.21 0.61–3.05 0.84–1.63	0.033 0.001 0.451 0.343	1.45 2.62 0.98 1.20 1	0.53–3.94 1.20–5.70 0.39–2.47 0.76–1.88	0.466 0.015* 0.962 0.430
Confident that I can stay at home easily to prevent COVID-19						
Strongly disagree Disagree Neither agree or disagree Agree Strongly agree	1.94 1.05 0.95 1.13 1	1.14–3.32 0.65–1.68 0.55–1.63 0.73–1.75	0.015 0.854 0.852 0.577	1.50 0.73 0.71 0.99 1	0.71–3.17 0.39–1.36 0.36–1.39 0.57–1.70	0.283 0.326 0.316 0.958
Confident that I can avoid crowed places and close contact						
Strongly disagree Disagree Neither agree or disagree Agree Strongly agree	1.54 1.36 1.26 1.10 1	0.64–3.73 0.77–2.40 0.75–2.13 0.78–1.56	0.339 0.291 0.382 0.578	0.91 0.76 1.14 1.05 1	0.28–2.96 0.34–1.69 0.56–2.32 0.65–1.71	0.879 0.500 0.711 0.843
Always cover cough using the bend of my elbow						
Strongly disagree Disagree Neither agree or disagree Agree Strongly agree	2.20 1.71 1.38 0.92 1	0.48–10.06 0.85–3.47 0.72–2.64 0.66–1.27	0.308 0.135 0.326 0.604	0.98 1.11 1.26 1.07 1	0.09–10.69 0.41–2.99 0.52–3.05 0.65–1.76	0.985 0.837 0.606 0.778
Avoid touching my eyes nose and mouth to prevent infection of COVID-19						
Strongly disagree Disagree Neither agree or disagree Agree Strongly agree	3.92 1.04 0.95 0.89 1	1.21–12.72 0.48–2.24 0.54–1.66 0.64–1.24	0.023 0.928 0.851 0.493	3.34 0.76 0.75 0.75	0.49–22.56 0.27–2.10 0.33–1.70 0.44–1.27	0.217 0.594 0.494 0.281
Maintain at least 2-m distance between myself and any other individuals						
Strongly disagree Disagree Neither agree or disagree Agree Strongly agree	1.36 1.16 1.43 0.91 1	0.45–4.06 0.67–2.02 0.86–2.36 0.64–1.31	0.587 0.585 0.165 0.629	0.73 0.66 1.28 0.79 1	0.16–3.33 0.31–1.41 0.66–2.49 0.48–1.32	0.684 0.287 0.470 0.374
Believing that COVID-19 is extremely harmful						
Strongly disagree Disagree Neither agree or disagree Agree Strongly agree	1.18 0.43 0.83 0.71 1	0.66–2.11 0.22–0.85 0.44–1.55 0.51–0.99	0.571 0.015 0.552 0.043	1.38 0.55 0.70 0.70 1	0.71–2.68 0.26–1.14 0.34–1.42 0.48–1.02	0.346 0.107 0.323 0.064
Have the skill to follow recommended handwashing practices to prevent COVID-19 infection						
Strongly disagree Disagree Neither agree or disagree Agree Strongly agree	1.03 8.01 2.29 1.32 1	0.28–3.81 2.04–31.49 0.63–8.25 0.98–1.79	0.964 0.003 0.205 0.070	0.81 5.39 2.19 1.14 1	0.06–10.47 1.17–24.87 0.45–10.61 0.72–1.81	0.873 0.031* 0.331 0.569

Statistically significant values were shown in asterisk (*) at *p*-value less than 0.05

**Fig. 3:**
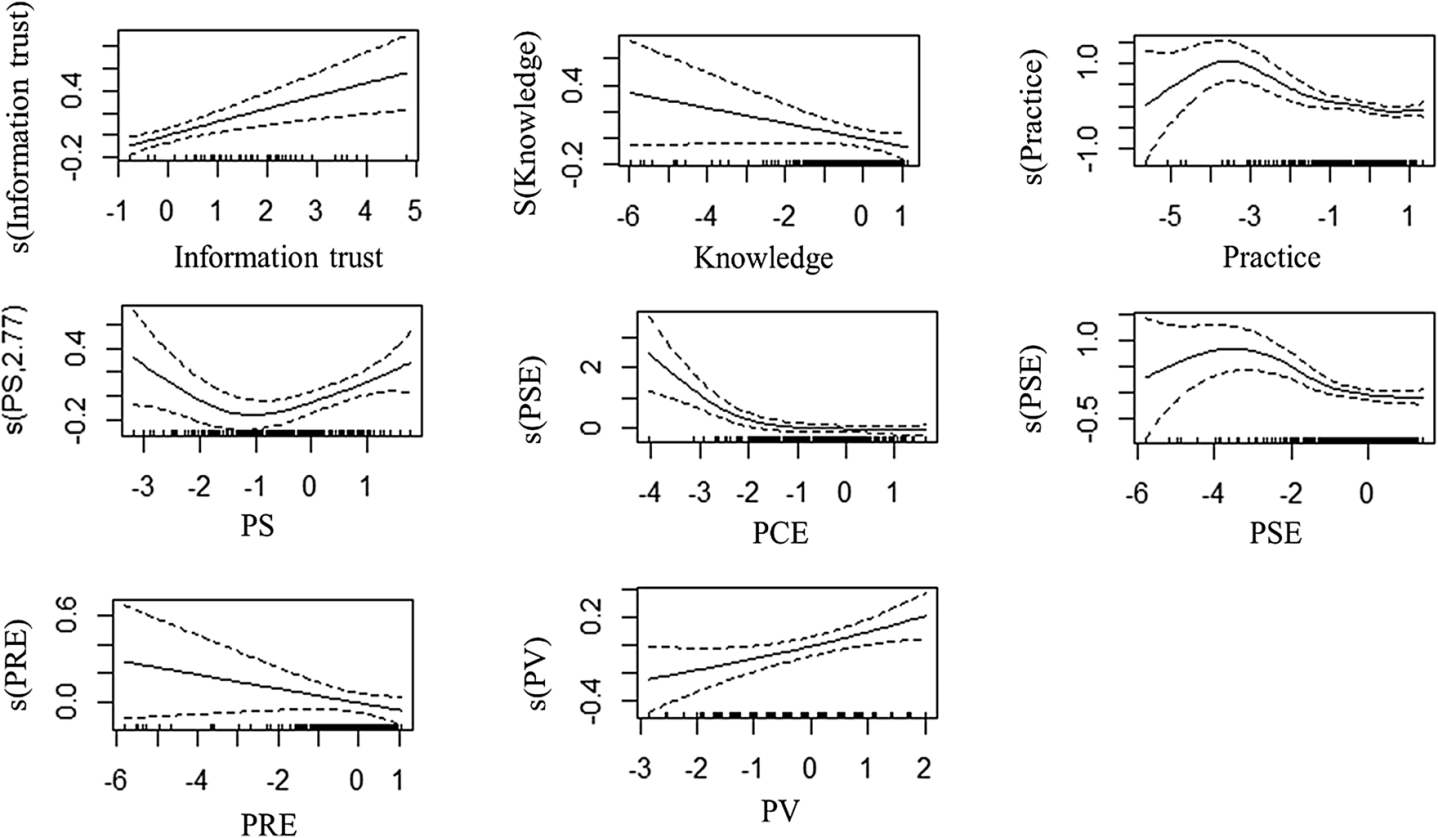
GAM model of psychological distress against different predicting variables related to COVID-19 among the literate community of Ethiopia, 2020. *PRE* perceived response efficacy, *PSE* perceived self-efficacy—personal level, *PV* perceived vulnerability, *PCE* perceived collective efficacy, *PS* perceived severity/seriousness

In addition to the logistic regression, generalized additive model (GAM) was used to predict the psychological distress among the Ethiopian communities. The GAM model demonstrated that psychological distress was significantly (*p*-value < 0.01) predicted by level of trust on information, practice on coronavirus prevention, perceived severity, perceived collective efficacy, and perceived vulnerability of the participants. Interestingly, those who are practicing coronavirus infection prevention activities, such as social distancing, handwashing, staying at home, and avoiding crowded places, had significantly less psychological distress. We also have identified that, when perceived collective efficacy increases, psychosocial distress decrease. Conversely, those who had the highest score of information trust and the highest score perceived vulnerability about coronavirus had the highest score of psychological distress (Fig. 3).

## Discussion

The purpose of this study was to explore the psychological distress among the Educated Ethiopian population during the COVID-19 pandemic and identify the associated factors. In Ethiopia, 25.5% of participants had high psychological distress during the COVID-19 pandemic (45.1%, had low psychological distress, 29.4% had moderate psychological distress, 17.6% had high psychological distress, and 7.3% had very high psychological distress). These rates of prevalence are considerably lower than those reported from China, where about 35% of the respondents experienced high psychological distress [17]. On the other side, our study finding is closely similar to the psychosocial distress prevalence (29.3%) of Italy [18]. The finding disparities might be due to the socio-cultural differences and measurement tools used to assess the psychological distress. Multivariable analysis revealed that those who get information from social media were more likely to have psychological distress. This finding is in agreement with the previous study, where social media exposures were associated with anxiety[19].

The possible reason might be during the COVID-19 pandemic, misinformation, myths about the COVID-19 pandemic have bombarded through social media, which strengthened groundless stress about COVID-19 among the population [20]. Trusting information coming from different sources might expose people to metal stress. Hence the use of information only from trusted and authorized source could alleviate the problem. Besides, many people state their negative feelings, such as fear, worry, nervousness, anxiety on social media, which can lead to transfer of emotional states to others via emotional contagion, leading people to have similar emotions without their awareness [21]. So caution is necessary concerning getting information about COVID-19 on social media and better use of information delivered by WHO’s ‘infodemics’ team [6].

Our finding revealed that participants who do not wash their hands frequently with soap and water, not having the resource (water and soap) to wash their hands, and those who have no the skill to follow recommended handwashing practices, had higher odds of psychological distress. The absence of hand hygiene resources and not washing their hands inadequately could have made individuals fear contracting the COVID-19 infection, which is associated with high psychological distress. This emphasizes the importance of compliance with infection prevention and control practices of the WHO-5 hand hygiene campaign—consisting of five components, namely system change, training and education, observation and feedback, reminders, and a safety climate—found it to be effective in improving hand hygiene in the community, and found that compliance was further improved by adding behavioral interventions such as goal setting, reward incentives, and accountability [22, 23].

Furthermore, during the COVID-19 pandemic, when the need for hand hygiene supply is considerably increased, sustaining the required supplies is critically essential to maintain frequent hand hygiene. These findings should inform strategies designed to increase supplies needed for infection prevention and control and to influence the behavioral factors of compliance with hand hygiene practices.

We found a significant association between age and psychological distress. This is consistent with a study conducted in Australia during the influenza epidemic [24]. The possible reason may be that young and middle-aged adults were most at risk and were coping less well with the consequences [23] and they are also less likely to be resilient or skilful, mostly when it comes to handling a difficulty. Also, there are varying observations about how age affects psychological distress with a lack of consistent results across studies. This has been largely attributed to different patterns of exposure to risk factors across age groups in various studies [25, 26].

In general, our study identified considerable psychological distress among the communities during the COVID-19 pandemic that needs the attention of health service institutions to intervene in the problem. A psychological crisis intervention plan needs to be developed in a cultural context and health education for the Ethiopian population on awareness creation and how to reduce the psychological impact of COVID-19-induced distress. Besides, psychological counselors/counseling psychologists should regularly visit people with psychological distress to listen to their stories for their stress and provide support. Therefore, it is a timely need for pertinent stakeholders to support the Ethiopian public health care system to introduce novel approaches to generate financially sustainable programs for the prevention of psychological distress among the Ethiopian population through a group of well-trained psychologists.

Our study has limitations. We collect the data after 2 months of the COVID-19 outbreak. Therefore, the period of exposure to the COVID-19 was short. We could only study the acute psychological impact and might not be generalized to sub-acute and long-term psychological complications if the outbreak continues. This study was a cross-sectional study, not able to determine cause-and-effect relationships between these variables. Besides, our study covers only communities who could read and write in English and had internet access.

## Conclusion

This study indicates that, in the literate community of Ethiopians, the prevalence of high psychological distress was substantial. Those who have alternative information sources and trust the information need special attention and intervention. To reduce the psychosocial distress, promotion of practicing the preventive measures also could enhance their confidence of not contracting the disease. In conclusion, we suggest that focussing only on the COVID-19 prevention and treatment is not sufficient to overcome the problems related to the pandemic. Development of an intervention plan to intervene in the psychological distress in the population, mainly targeting those groups who got information from social media, young and middle-aged adults, and those who do not adequately practice infection prevention and control measures to prevent COVID-19 infection. This study was made in the early onset of the disease and follow-up studies could be vital in determining the dynamics of psychosocial distress, more specifically after the introduction of COVID-19 vaccination.

## References

[1] Hawryluck L, Gold WL, Robinson S, Pogorski S, Galea S, Styra R (2004). SARS Control and Psychological Effects of Quarantine, Toronto. Canada Emerg Infect Dis.

[2] WHO. Statement on the second meeting of the International Health Regulations (2005) Emergency Committee regarding the outbreak of novel coronavirus (2019-nCoV). Geneva, Switzerland; 2005.

[3] Mahase E. China coronavirus: WHO declares international emergency as death toll exceeds 200. BMJ. 2020;m408. 10.1136/bmj.m408 32005727

[4] Guo Y-R, Cao Q-D, Hong Z-S, Tan Y-Y, Chen S-D, Jin H-J (2020). The origin, transmission and clinical therapies on coronavirus disease 2019 (COVID-19) outbreak—an update on the status. Military Med Res.

[5] Jindo T, Kai Y, Kitano N, Tsunoda K, Nagamatsu T, Arao T (2020). Relationship of workplace exercise with work engagement and psychological distress in employees: a cross-sectional study from the MYLS study. Prevent Med Rep.

[6] WHO. WHO Director-General’s opening remarks at the media briefing on COVID-19 - 11 March 2020; 2020. https://www.who.int/dg/speeches/detail/who-director-general-s-opening-remarks-at-the-media-briefing-on-covid-19---11-march-2020. Accessed 25 May 2020.

[7] Niu Y, Xu F (2020). Deciphering the power of isolation in controlling COVID-19 outbreaks. Lancet Glob Health.

[8] Brooks SK, Webster RK, Smith LE, Woodland L, Wessely S, Greenberg N (2020). The psychological impact of quarantine and how to reduce it: rapid review of the evidence. The Lancet.

[9] Tanskanen J, Anttila T (2016). A prospective study of social isolation, loneliness, and mortality in Finland. Am J Public Health.

[10] Barbisch D, Koenig KL, Shih F-Y (2015). Is There a case for quarantine? Perspectives from SARS to Ebola. Disaster Med Public Health Prep.

[11] Miles SH (2015). Kaci Hickox: public health and the politics of fear. Am J Bioeth.

[12] Pellecchia U, Crestani R, Decroo T, Van den Bergh R, Al-Kourdi Y. Social Consequences of Ebola Containment Measures in Liberia. In: Braunstein LA, editor. PLoS ONE. 2015;10:e0143036. 10.1371/journal.pone.0143036 PMC4674104 26650630

[13] Mihashi M, Otsubo Y, Yinjuan X, Nagatomi K, Hoshiko M, Ishitake T (2009). Predictive factors of psychological disorder development during recovery following SARS outbreak. Health Psychol.

[14] Kessler RC, Andrews G, Colpe LJ, Hiripi E, Mroczek DK, Normand S-LT (2002). Short screening scales to monitor population prevalences and trends in non-specific psychological distress. Psychol Med.

[15] Andrews G, Slade T (2001). Interpreting scores on the Kessler Psychological Distress Scale (K10). Aust N Z J Public Health.

[16] Australian Bureau of Statistics. Use of the Kessler psychological distress scale in ABS health surveys, Australia, 2001. Report No.: Information Paper 4817.0.55.001. Australia: Australian Bureau of Statistics; 2003. https://www.abs.gov.au/ausstats/abs@.nsf/ProductsbyReleaseDate/4D5BD324FE8B415FCA2579D500161D57. Accessed 25 May 2020.

[17] Qiu J, Shen B, Zhao M, Wang Z, Xie B, Xu Y (2020). A nationwide survey of psychological distress among Chinese people in the COVID-19 epidemic: implications and policy recommendations. Gen Psych.

[18] Eskin M, Sun J-M, Abuidhail J, Yoshimasu K, Kujan O, Janghorbani M (2016). Suicidal behavior and psychological distress in University students: a 12-nation study. Arch Suicide Res.

[19] Neria Y, Sullivan GM (2011). Understanding the mental health effects of indirect exposure to mass trauma through the media. JAMA.

[20] Xinhua. Bat soup, biolab, crazy numbers...Misinformation “infodemic” on novel coronavirus exposed. China; 2020. http://www.xinhuanet.com/english/2020-02/04/c_138755586.htm. Accessed 25 May 2020.

[21] Kramer ADI, Guillory JE, Hancock JT (2014). Experimental evidence of massive-scale emotional contagion through social networks. Proc Natl Acad Sci.

[22] Huis A, van Achterberg T, de Bruin M, Grol R, Schoonhoven L, Hulscher M (2012). A systematic review of hand hygiene improvement strategies: a behavioural approach. Implementation Sci.

[23] Luangasanatip N, Hongsuwan M, Limmathurotsakul D, Lubell Y, Lee AS, Harbarth S, et al. Comparative efficacy of interventions to promote hand hygiene in hospital: systematic review and network meta-analysis. BMJ. 2015;h3728. 10.1136/bmj.h3728 PMC4517539 26220070

[24] Taylor MR, Agho KE, Stevens GJ, Raphael B (2008). Factors influencing psychological distress during a disease epidemic: data from Australia’s first outbreak of equine influenza. BMC Public Health.

[25] Drapeau A, Marchand A, Forest C (2014). Gender differences in the age-cohort distribution of psychological distress in Canadian adults: findings from a national longitudinal survey. BMC Psychol.

[26] Jorm AF, Windsor TD, Dear KBG, Anstey KJ, Christensen H, Rodgers B (2005). Age group differences in psychological distress: the role of psychosocial risk factors that vary with age. Psychol Med.

